# IGF1 Signaling Regulates Neuropeptide Expression in Hypothalamic Neurons Under Physiological and Pathological Conditions

**DOI:** 10.1210/endocr/bqaf051

**Published:** 2025-03-19

**Authors:** Wenyuan He, Neruja Loganathan, Denise D Belsham

**Affiliations:** Department of Physiology, University of Toronto, Toronto, ON, Canada M5S 1A8; Department of Physiology, University of Toronto, Toronto, ON, Canada M5S 1A8; Department of Physiology, University of Toronto, Toronto, ON, Canada M5S 1A8; Department of Medicine, University of Toronto, Toronto, ON, Canada M5S 1A8; Department of Obstetrics and Gynaecology, University of Toronto, Toronto, ON, Canada M5S 1A8

**Keywords:** hypothalamus, IGF1, insulin, hyperinsulinemia, IGF1 resistance, neuropeptides

## Abstract

Insulin-like growth factor 1 (IGF1) plays a critical role in metabolism and aging, but its role in the brain remains unclear. This study examined whether hypothalamic neurons respond to IGF1 and how its actions are modulated. RT-qPCR and single-cell RNA sequencing indicated that *Igf1r* mRNA is expressed in neuropeptide Y/Agouti-related peptide (NPY/AgRP) neurons but has higher expression in pro-opiomelanocortin (POMC) neurons. IGF1 binding proteins *Igfbp3* and *Igfbp5* were significantly expressed, whereby *Igfbp5* levels were modulated by fasting, nutrient availability, and circadian rhythms, implying that IGF1 signaling can be controlled by multiple mechanisms. In mouse and human models, IGF1 regulated *Agrp*, *Npy*, *Pomc*, *Cartpt*, *Spx*, *Gal*, and *Fam237b* expression, producing an overall anorexigenic profile. Hyperinsulinemia induced IGF1 resistance, accompanied by reduced IGF1R protein, as well as *Igf1r* and *Irs2* mRNA expression via over-activation of phosphoinositide 3-kinase/forkhead box O1 (PI3K-FOXO1) signaling. Thus, hypothalamic neurons respond to IGF1 under physiological conditions, and hyperinsulinemia is a novel mechanism that drives cellular IGF1 resistance.

Approximately 16% of the global population is obese (1), and about 6.3% has type 2 diabetes (2). Obesity contributes to the development of multiple comorbidities, including type 2 diabetes, cardiovascular diseases, and certain types of cancer, leading to a significant reduction in both the length and quality of life. A contributing factor to the development of obesity is a dysregulation in the central control of appetite and body weight. The arcuate nucleus of the hypothalamus (ARC) contains neuropeptide Y/agouti-related peptide (NPY/AgRP) and pro-opiomelanocortin (POMC) neuronal populations that integrate nutrients and hormonal signals, such as insulin, ghrelin, and leptin, to control energy intake, expenditure, and metabolism (3, 4). In addition, hypothalamic neurons are controlled by an intrinsic molecular clock composed of the central oscillator BMAL1:CLOCK and PER2:CRY, which form a transcriptional translational feedback loop that governs genome expression and ultimately feeding patterns in line with day-night rhythms (4-6).

The ARC also has a more permeable blood-brain barrier and is exposed to higher levels of circulating hormones compared to the rest of the brain (7, 8). Several hormones act on ARC neurons to convey the energy status of the body. These hormones share 2 important characteristics: First, they reach the hypothalamus, sometimes being specifically transported via mechanisms such as transcytosis. Second, these hormones alter neuropeptide expression or the electrical activity of NPY/AgRP and POMC neurons (9, 10). For example, insulin is transported to the brain and its central action has been demonstrated to regulate food intake in both animal and human models (11). Insulin has a paralog with high sequence homology, insulin-like growth factor 1 (IGF1). IGF1 is primarily produced in the liver and plays a crucial role in growth and development by mediating the effects of growth hormone. Its receptor, the insulin-like growth factor 1 receptor (IGF1R), also shares high homology with the insulin receptor (INSR), and they both share several downstream signaling molecules. However, IGF1 and insulin also produce distinct transcriptomic effects (12). A key difference between IGF1 and insulin is that IGF1 availability and signaling is regulated by a set of 6 IGF binding proteins (IGFBP1-6). The primary function of the IGFBPs is to sequester IGF1 and prevent it from binding IGF1R, thereby reducing IGF1 signaling.

The role of IGF1 in centrally regulating appetite and peripheral metabolism remains controversial, with limited recognition of IGF1 as a significant factor (13). Heterozygous deletion of IGF1R in the brain using Nestin-Cre resulted in reduced body weight, increased adiposity, hyperleptinemia, and glucose intolerance (14). However, central administration of IGF1 has produced conflicting results. Similar to insulin, central IGF1 administration improved glucose tolerance and insulin sensitivity in mice (15); however, other studies have shown that central IGF1 increased, decreased, or had no effect on food intake in rats (15, 16). Central IGF1 administration also promoted anorexia in chicks (17, 18). Furthermore, several other questions regarding IGF1 signaling in the brain remain unanswered. First, it is unclear whether neurons in the ARC express sufficient levels of IGF1R and can respond to IGF1. Second, baseline levels of IGF1 are in the range of nanomolar in the brain, as contrasted to insulin being in the range of picomolar, so IGF1 administration may not represent a physiological approach, because a physiological spike of total IGF1 in the brain may never occur (19, 20). Although fasting reduces circulating IGF1 in humans, whether this change can be sensed in the brain and whether fasting or metabolic stress can alter IGF1 signaling in the hypothalamus remains unclear.

In this study, we used single-cell RNA sequencing datasets and cell models of hypothalamic NPY/AgRP and POMC neurons, including human induced pluripotent stem cell (iPSC)-derived neurons, mouse primary neurons, and mouse immortalized cell lines, to show that a population of hypothalamic NPY/AgRP and POMC neurons expresses *Igf1r*, *Igfbp3*, and *Igfbp5*. They are also significantly expressed in the hypothalamus and in ARC neurons, whereby fasting and serum starvation modulated the expression of *Igfbp3* and *Igfbp5* in both cultured hypothalamic neurons and ARC neurons in vivo. Furthermore, *Igfbp5* mRNA exhibited circadian expression in the mouse hypothalamus and in cultured hypothalamic neurons, which is significantly abrogated in neurons lacking BMAL1. These results provide evidence that hypothalamic NPY/AgRP and POMC neurons have the capacity to respond to IGF1 and suggest that IGF1 signaling may be modulated at the IGFBP level in response to energy status and by circadian rhythms. IGF1 treatment induced an overall anorexigenic pattern in neuropeptide mRNA levels. Finally, metabolic stress in the form of hyperinsulinemia promoted cellular IGF1 resistance via downregulation of *Igf1r* mRNA and protein, through a phosphoinositide 3-kinase/forkhead box O1 (PI3K-FOXO1)-mediated transcriptional mechanism. Taken together, these results indicate that hypothalamic neurons can respond to IGF1 and receive variable levels of IGF1 input, leading to changes in neuropeptide expression. Hyperinsulinemia is also identified as a novel mechanism underlying tissue-level IGF1 resistance observed in human obesity and animal models of the disease. Overall, we describe the regulation of IGF1 signal modulators and highlight the anorexigenic role of IGF1 in the ARC hypothalamus, and we provided evidence that hyperinsulinemia induces cellular IGF1 resistance through reduced IGF1R levels in a PI3K-FOXO1–dependent manner. This IGF1 resistance may, in turn, contribute to the development of obesity and its related comorbidities.

## Materials and Methods

### Hypothalamic Cell Lines

The clonal, immortalized hypothalamic mHypoE-46 neuronal cell line (male), representative of an ARC NPY/AgRP neuron (male), was generated and extensively characterized as previously described (21-23). mHypoA-POMC/GFP-1 (male) were previously generated from C57BL/6J-Tg (POMC-EGFP) mouse and characterized (24). The mHypoA-BMAL1-WT/F8 (female) and mHypoA-BMAL1-KO/F2 (female) clonal cell lines were derived from the female heterogeneous hypothalamic wild-type (WT) and knockout (KO) lines, respectively, by single-cell subcloning via serial dilution. In brief, hypothalami were collected from BMAL1^+/+^ (wild-type littermate controls; WT) and BMAL1^−/−^ (knockout; KO) C57BL/6J mice (both males and females), bred from BMAL1^+/−^ mice obtained from Jackson Laboratories. The hypothalami were triturated, placed into primary culture, treated with ciliary neurotrophic factor to induce neuronal proliferation, and immortalized via retroviral transfer of SV-40 T-antigen. Absence of BMAL1 protein expression in the mHypoA-BMAL1-KO cell lines was confirmed by Western blot analysis (25). Both cell lines displayed similar gene expression profiles and represent *Npy* + and *Agrp* + neuronal populations.

All cell lines were cultured in low-glucose (5.5 mM) Dulbecco's Modified Eagle Medium (DMEM; MilliporeSigma) containing 5% fetal bovine serum (FBS; Gibco) and 1% penicillin-streptomycin (Gibco). Neurons were regularly passaged in 100 mm tissue culture plates (Sarstedt) using 1× Trypsin/EDTA (Gibco). Twenty-four hours prior to all experiments, neurons were passaged into 60- or 100-mm tissue culture plates (Sarstedt).

### Reagent Preparation

PD 0325901 (Tocris Bioscience, 4192) is a selective MEK inhibitor, while LY294002-HCl (Tocris Bioscience, 1130) and Wortmannin (Tocris Bioscience, 1231) are PI3K inhibitors. Triacsin-C is an inhibitor of long fatty acyl-CoA synthetase (Abcam, Ab141888); all 4 inhibitors were prepared as stock solutions in dimethyl sulfoxide (DMSO) at concentrations 1000 times higher than the intended treatment concentration. Corresponding vehicle groups were treated with an equivalent concentration of DMSO. Insulin Aspart, Novorapid Penfill (Novo Nordisk, DIN 02244353) was diluted to a concentration 1000 times higher than the treatment concentration for the stock solution in 1× phosphate-buffered saline (PBS). Recombinant human IGF-I/IGF-1 protein, CF (Bio-Techne, #291-G1), and recombinant mouse IGF-I/IGF-1 protein, CF (Bio-Techne, #791-MG), were dissolved in PBS to prepare stock solutions at concentrations 1000-fold higher than the intended treatment concentrations. To examine the effects of insulin and IGF-1 on transcription, cells were cultured in serum-free DMEM for 3 hours, followed by 6 hours of treatment with the respective proteins in serum-free DMEM. To induce cellular insulin resistance, neurons were treated with 100nM insulin for 24 hours in DMEM supplemented with 5% FBS and 1% penicillin-streptomycin (P/S). For the insulin-induced IGF1 resistance experiments, the above paradigm was followed by incubation in serum-free media for 1 hour and subsequent treatment with 10nM IGF1 for 6 hours.

### Analysis of Rhythmic Gene Expression

For time-course experiments, mHypoA-BMAL1-WT/F8 and mHypoA-BMAL1-KO/F2 cells were plated into 60 mm cell culture plates and cultured for 24 hours until they reached 50% to 60% confluency. The cells were then cultured in serum-free DMEM for 120 minutes, followed by treatment with forskolin (20μM) for 30 minutes in 2.5 mL of media to synchronize the cells. After treatment, the media was replaced with fresh DMEM containing 5% FBS. Phosphate-buffered saline washes were performed between changing from serum-containing to serum-free media, and again when transitioning from forskolin treatment to the 5% FBS treatment. Cells were harvested every 6 hours.

### Generation and Characterization of Human iPSC-Derived Hypothalamic-Like Neurons

The use of the human stem cell line was approved by the University of Toronto Research Ethics Board. Human BJ induced pluripotent stem cells (iPSCs) were cultured in 6-well dishes coated with Geltrex™ LDEV-Free Reduced Growth Factor Basement Membrane Matrix (1:200 dilution) (Thermo Fisher Scientific, A1413202) in StemMACS™ iPS-Brew XF medium (Miltenyi Biotec, 130-104-368) supplemented with the ROCK inhibitor Y-27632 (Cayman Chemicals, 10005583). Once confluent, the cells were differentiated using differentiation media following previously established methods (6, 7). Briefly, the cells were differentiated into neuroectoderm cells via dual SMAD inhibition using LDN193189 (1μM, Cayman Chemicals, 11802) and SB431542 (10μM, Cayman Chemicals, 13031) for 48 hours. From day 2 to day 9, the cells were directed toward a ventral diencephalon forebrain cell fate using Sonic hedgehog activation—smoothened agonist (SAG, 1μM, Tocris Bioscience, 4366) and purmorphamine (PMN, 1μM, Tocris Bioscience, 4551)—and WNT signaling inhibition with IWR1-endo (10μM, Cayman Chemicals, 13659). During this period (day 2 to day 9), the cells are exposed to these inhibitors in addition to LDN193189 and SB431542. From day 9 to day 16, 10μM DAPT (Cayman Chemicals, 13197), a gamma secretase inhibitor, was used to allow the cells to exit the cell cycle in the presence of 10nM retinoic acid (Cayman Chemicals, 11017). On day 13, the cells were trypsinized using TrypLE™ Express Enzyme (1X), no phenol red (Gibco, 12604013), passed through a 40-μm filter, and diluted at ratios of 1:3 or 1:4 onto new 6-well plates coated with Geltrex (1:50 dilution) in differentiation media containing retinoic acid. The media was changed daily during this process. From day 16 onwards, the cells matured with the addition of 10 ng/mL brain-derived neurotrophic factor (BDNF, Miltenyi Biotec, 130-096-286) to form hypothalamic-like neurons. The media was replaced every 2 to 3 days until day 21, when the cells were used for treatment. The differentiation media used up to day 13 consisted of DMEM/F-12, GlutaMAX™ Supplement (Gibco, 10565018) supplemented with 1× B-27™ Supplement (50X), minus vitamin A (Gibco, 12587010), 1× N-2 Supplement (100X) (Gibco, 17502048), 1× non-essential amino acids (NEAA, Gibco, 11140050), 1× GlutaMAX™ Supplement (Gibco, 35050061), 1× Anti-Anti (Antibiotic-Antimycotic) Solution (Gibco, 15240062), 0.15% (w/v) D-(+)-Glucose, Anhydrous (MilliporeSigma, G7021), and 200nM of L-Ascorbic Acid (Vitamin C) (MilliporeSigma, A4544). From day 13 onwards, maturation media was used, comprising DMEM/F-12, GlutaMAX™ Supplement (Gibco, 10565018) with 1× B-27™ Supplement (50X), minus insulin (Gibco, A1895601), 1× N-2 Supplement (100X), 1× NEAA, 1× GlutaMAX, 1× Anti-Anti (Antibiotic-Antimycotic) Solution, 0.15% (w/v) glucose, and 200nM ascorbic acid. Cells were treated in DMEM/F-12 media, containing approximately 5mM glucose and supplemented with 1× B-27™ Supplement (50X), minus insulin, 1× NEAA, 1× GlutaMAX, and 1× Anti-Anti (Antibiotic-Antimycotic) Solution.

### Hypothalamic Primary Culture

Animal studies were conducted in accordance with the Ontario Animals for Research Act and the federal Canadian Council on Animal Care guidelines. All procedures were approved by the University of Toronto Animal Care Committee. Eight-week-old male CD-1^®^ IGS mice (Charles River, CRL: 022) were euthanized using carbon dioxide (CO_2_) inhalation followed by cervical dislocation. The hypothalami were dissected, washed with 1× PBS, trypsinized, and triturated to obtain a cell suspension, which was plated on poly-L-lysine-coated plates. Primary hypothalamic cells were cultured in Neurobasal A Medium (Gibco, 10888022) containing 1× GlutaMAX™ Supplement (Gibco, 35050061), 1× B-27™ Supplement (50X), minus vitamin A (Gibco, 12587010), 10% FBS (Gibco, 26140079), 5% normal horse serum, and 1% penicillin-streptomycin (Gibco, 15140122). After 1 week, the growth medium was removed, and the primary culture was treated with 100μM insulin Aspart, Novorapid Penfill (Novo Nordisk, DIN 02244353) for 24 hours, as described above.

### RNA Isolation, cDNA Synthesis, and Reverse Transcription–Quantitative Polymerase Chain Reaction

After treatment, cells were washed with 1× PBS, and total RNA was extracted using the Total RNA Purification Kit (Norgen Biotek Corp., 37500) and anhydrous ethanol (Commercial Alcohols, N/A). Complementary DNA (cDNA) was synthesized from 1000 ng of total RNA using the High-Capacity cDNA Reverse Transcription Kit (Applied Biosystems, Thermo Fisher Scientific, 4368814). Reverse transcription–quantitative polymerase chain reaction (RT-qPCR) was conducted using gene-specific primers (Table 1) and PowerTrack SYBR Green Master Mix (Thermo Fisher Scientific, A46109), with cDNA samples run in triplicate. The qPCR was performed on the QuantStudio 5 Real-Time PCR System (Thermo Fisher Scientific). The relative mRNA expression was determined using the ΔΔCt method and normalized to the stable reference genes 60S ribosomal protein L7 (Rpl7), RNAse III endonuclease Drosha (Drosha), or histone H3A, as indicated in each figure. For each experimental replicate, the average Ct values of the triplicates were used, and relative expression levels were calculated by comparing the treated groups to the vehicle control.

**Table 1.** List of primers for RT-qPCR

Gene	Primer sequence (5′-3′)	Amplicon size (bp)
*Rpl7*	F: TCGCAGAGTTGAAGGTGAAG R: GCCTGTACTCCTTGTGATAGTG	114
*Npy*	F: CAGAAAACGCCCCCAGAA R: AAAAGTCGGGAGAACAAGTTTCATT	77
*Agrp*	F: CGGAGGTGCTAGATCCACAGA R: AGGACTCGTGCAGCCTTACAC	69
*Atf4*	F: GGAGCAAAACAAGACAGC R: TTGCCTTACGGACCTCTTCT	179
*Pomc*	F: CCCGCCCAAGGACAAGCGTT R: CTGGCCCTTCTTGTGCGCGT	112
*Cartpt*	F: AAGTCCCCATGTGTGACGC R: CAGTCACACAGCTTCCCGAT	73
*Fam237b (Gm8773)*	F: TCACCAGCGTAGCACAAAGG R: TACAGCAGAGCCCATCCCAAA	155
*Spx*	F: CGCCTCCAGAAAGACGAAAC R: AATTCCCTCCTTCATCTGCACC	114
*hRPL7*	F: TGGCAAGAAAAGCTGGCAAC R: ACCTTTCGAACCTTTGGGCT	103
*hHIS3A*	F: GCAAGAGTGCGCCCTCTACTG R: GGCCTCACTTGCCTCGTGCAA	218
*hDROSHA*	F: GGAGCTGGAGTGGCAGAAAT R: AGAGCTTGGTTTCGTCCCAG	76
*hAGRP*	F: GCCTTGGCAGAGGTACTAGA R: AGGACTCATGCAGCCTTACG	76
*hPOMC*	F: GCAACCTGCTGGAGTGCAT R: CAGTCTTCGCCCGCTGAG	217
*hNPY*	F: ATCAACCTCATCACCAGGCAG R: CACCACATTGCAGGGTCTTC	128
*Bmal1*	F: GGGAGGCCCACAGTCAGATT R: GTACCAAAGAAGCCAATTCATCAA	78
*Per2*	F: TCATCATTGGGAGGCACAAA R: GCATCAGTAGCCGGTGGATT	135
*Igfbp3*	F: GTTCCATCCACTCCATGCCA R: CGGCAGGGACCGTATTCTG	150
*Igfbp5*	F: GCGAGCAAACCAAGATAGAGAGAG R: GGAAATGCGAGTGTGCTTGG	116
*Igf1r*	F: GCACCAATGCTTCAGTCCCT R: TGGGTATTTTGTCTTTGGAGCAGT	195
*Insr*	F: TCACCTGAGTCCCTGAAGG R: GCATCAGGTCAGTGAGTCTC	199
*hIGF1R*	F: AGTATGGAGGGGCCAAGCTA R: ACGACCCATTCCCAGAGAGA	87
*hINSR*	F: ACATGGAGAATGTGCCCCTG R: AAGGATTGGACCGAGGCAAG	177
*hIRS1*	F: GGTGGATGACTCTGTGGTGG R: GGACGCTGATGGGGTTAGAG	131
*hIRS2*	F: CTCTGCCTCGCTGGATGAAT R: GTCTCCGTAGTCCTCTGGGT	130

### Western Blotting

Protein was harvested using 1× cell lysis buffer (Cell Signaling Technology, Inc., 9803) containing 1 mM phenylmethylsulfonyl fluoride (PMSF, MilliporeSigma, P7626), 1% protease inhibitor cocktail (MilliporeSigma, P8340), and 1% phosphatase inhibitor cocktail 2 (MilliporeSigma, P5726). The protein concentration was quantified using the Pierce™ BCA Protein Assay Kit (Thermo Fisher Scientific, 23225). Twenty micrograms (µg) of total protein was separated on 12% SDS-polyacrylamide gels (custom-prepared) and transferred onto polyvinylidene difluoride (PVDF) membranes (Bio-Rad). Membranes were blocked in 5% non-fat dry milk (Bio-Rad, 1706404) in Tris-buffered saline with Tween 20 (TBS-T, custom-prepared) for 1 hour, followed by incubation with primary antibodies overnight at 4 °C for approximately 16 to 20 hours. The primary antibodies (Table 2): IGF1R-β (Cell Signaling Technology, Inc., 3027), alpha-tubulin (Cell Signaling Technology, Inc., 2144), and INSR-β (Cell Signaling Technology, Inc., 3025) were diluted 1:1000 in 5% non-fat dry milk in TBS-T. Membranes were washed 3 times for 10 minutes each with TBS-T prior to incubation with the secondary antibody anti-rabbit antibody (Cell Signaling Technology, Inc., 7074; 1:7500 dilution in 5% milk in TBS-T) for 1 hour. Membranes were then washed 6 times for 5 minutes each prior to imaging, which was performed using the SignalFire ECL Reagent (Cell Signaling Technology, Inc., 6883) on an iBright™ FL1500 or iBright™ FCL1500 imager. Alpha-tubulin was imaged on the same blot using the Restore PLUS Western Blot Stripping Buffer (Thermo Fisher Scientific, 46430) before probing the blot for subsequent proteins, according to the manufacturer's instructions. Protein density was quantified using iBright Analysis Software (version 5.2.1), with background-corrected signal values and minimal adjustments to automated lane and band selection. The quantified values were then normalized to the alpha-tubulin signal and to each biological replicate. To probe INSR and IGF1R, duplicate Western blots were run using the same sample at the same time and were imaged separately and normalized to alpha-tubulin on each blot. This approach was necessary because the β subunits of both proteins have highly similar molecular weights, and stripping does not remove all residual primary and secondary antibodies. The INSR-β and IGF1R-β antibodies used were previously demonstrated to show no cross-reactivity in INSR/IGF1R double knockout models with reintroduced INSR and IGF1R (26).

**Table 2.** List of antibodies for Western blots

Reagent or Resource	Source	Identifier
**Antibodies**		
IGF1R-β Primary Antibody	Cell Signaling Technology	3027 RRID:AB_2122378
α-Tubulin Primary Antibody	Cell Signaling Technology	2144 RRID:AB_2210548
INSR-β Primary Antibody	Cell Signaling Technology	3025 RRID:AB_2280448
Anti-Rabbit Secondary Antibody	Cell Signaling Technology	7074 RRID:AB_2099233
α-MSH Primary Antibody	Phoenix Pharmaceuticals	H-043-01 RRID:AB_10013604
Alexa Fluor 488 Donkey Anti-Rabbit Secondary Antibody	Thermo Fisher Scientific	A21206 RRID:AB_2535792

### Immunofluorescence

D13 cells were seeded onto 2-well chamber slides (Thermo Fisher Scientific, 177402) and differentiated into D21 cells as described above. After washing with PBS and fixing in 4% paraformaldehyde (Electron Microscopy Sciences, 15710) in PBS for 10 minutes, the cells were again washed in PBS and permeabilized with 0.2% Triton X-100 in PBS (MilliporeSigma, T8787). They were then blocked for 2 hours at room temperature with 5% bovine serum albumin (BSA) (MilliporeSigma, A9647) and 0.1% Triton X-100 in PBS, followed by incubation with the anti–alpha-melanocyte stimulating hormone (α-MSH) primary antibody (Phoenix Pharmaceuticals, H-043-01) at 1:250 dilution in antibody dilution buffer (1% BSA and 0.1% Triton X-100 in PBS) for 2 hours at room temperature; control wells received only the antibody dilution buffer. After washing with the blocking buffer, the cells were incubated for 1 hour with Alexa Fluor 488 donkey anti-rabbit secondary antibody (Thermo Fisher Scientific, A21206) at 1:500 dilution in antibody dilution buffer, then washed, mounted with ProLong™ Gold Antifade Mountant containing DAPI (Thermo Fisher Scientific, P36935), and sealed. Cells were visualized using a Zeiss LSM700 confocal laser-scanning microscope at 20× magnification, and images were acquired with Zeiss Zen microscopy software.

### Bioinformatics Analysis of ChIP-Seq Data

Data processing was done following default settings of Cistrome data browser 3.0 (27). In brief, Cistrome Data Browser employs a Snakemake analysis pipeline for chromatin profiling reads. Reads are mapped using BWA (*bwa aln -q 5 -l 32 -k 2 -t 8 index FASTQ > sai; bwa samse index sai FASTQ > sam*), filtered and sorted with SAMtools (*samtools view-bS -t chromInfo_file.txt-q 1 sam > bam; samtools sort-m 4000000000 bam > bam_sort*), and read mapping statistics are generated (*samtools flagstat bam > bam.stat*). Peak calling is done using MACS2 (*macs2 callpeak --SPMR -B -q 0.01 --keep-dup 1 --extsize = 146 --nomodel -g hs -t bam -n test*). Datasets were selected if they passed 2 out of the 3 internal quality controls based on the number of peaks, fraction of reads in peaks, and fraction of peaks in DNAse sensitive regions. From left to right: **BMAL1:** GSM130167 (liver) (28), GSM1479716 (liver) (29), GSM2450976 (MEF, mouse embryonic fibroblast) (30), GSM3003975 (kidney) (31), GSM3003981 (heart) (31), GSM5044447 (liver). **FOXO1:** GSM1131775 (peripheral blood mononuclear cells) (32), GSM1480611 (CD4+ T cells) (33), GSM1874162 (uterus) (34), GSM3381273 (Liver) (35), GSM3831422 (islets of Langerhans) (36), GSM4278011 (circulatory organ) (37).

### Other Bioinformatics Analyses

Analysis of single-cell RNA sequencing data was performed using the CellxGene Discover platform on the HypoMap dataset (38). The arcuate region was identified using the authors' annotations, and genes with a normalized read count greater than 0 were considered positively detected (gene+). The transcriptomic analysis from Gallagher et al was conducted using the built-in Geo2R analysis tool with log-transformed data (39). The following gene probes were used: IGF1R (ENST00000333402_at), INSR (ENST00000341500_at), IRS1 (ENST00000305123_at), and IRS2 (ENST00000375856_at). This study also reanalyzed previously published RNA sequencing data (GSE68177), which involved bulk RNA sequencing of sorted arcuate *Npy*^hrGFP^ and *Pomc*^topazFP^ neurons obtained from respective male transgenic mice that were fasted for 24 hours (40). Reanalysis was performed using Limma Voom in R version 4.4.1 and RStudio 2024.04.2 + 764 (41).

### Statistical Analyses

Relative expression values for qPCR were batch-corrected by dividing each expression value by the intra-replicate average. This approach minimizes the effects of varying basal gene expression between replicates while maintaining the fold changes induced by the treatments. For succinct graphical representations, some data were normalized with respect to the control by dividing each value by the average of the corresponding control group (represented by the dotted line at y = 1). Statistical analyses were conducted after normalization, when applicable. Each replicate represented an independent cell culture preparation. Data were analyzed using GraphPad Prism version 10.1.2 (GraphPad Software) and are presented as the mean ± SEM. Data from each figure were analyzed using two-tailed *t* tests, one-way ANOVA, or two-way ANOVA with post hoc tests, as indicated in the figure legends. Changes in mRNA or protein levels were considered statistically significant if *P* < .05. Annotations are included in the respective figure legends.

## Results

### *Igf1r* Is Expressed in a Subset of NPY/AgRP and POMC Hypothalamic Neurons

**Figure 1.**
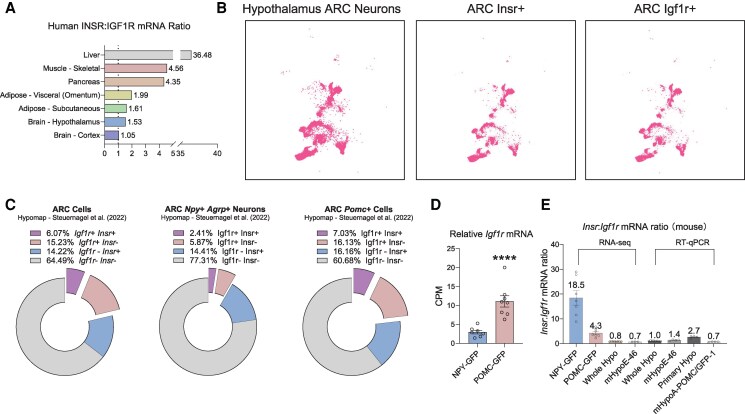
Expression pattern of *Igf1r* and *Insr* in arcuate hypothalamic neurons. (A) Relative human *INSR*-to-*IGF1R* mRNA ratios across various tissues in the GTEx dataset. (B) Single-cell sequencing data from HypoMap highlighting the ARC neurons expressing *Insr* and *Igf1r*. (C) Analysis of the data in (B), showing the percentages of IGF1R⁺ or INSR⁺ neurons among all ARC neurons, as well as among ARC NPY/AgRP⁺ or POMC⁺ subpopulations. (D) Proportion of IGF1R⁺ neurons, specifically the ARC INSR⁺/NPY/AgRP⁺ or ARC INSR⁺ /POMC⁺ subsets. (E) Relative *Insr*-to-*Igf1r* mRNA ratios as determined by RNA sequencing (FPKM) and RT-qPCR. Data are shown as mean ± SEM. Statistical significance in (E) was assessed using an unpaired *t* test. Sample sizes (N) range from 3 to 8, as indicated in each panel. *P < .05, **P < .01, ***P < .001, ****P < .0001.

We first questioned whether NPY/AgRP and POMC neurons have the capacity to respond to IGF1. While abundant *Igf1r* protein and mRNA could be detected in the hypothalamus, its distribution pattern specifically in ARC NPY/AgRP and POMC neurons remained unclear. We used the human genotype-tissue expression (GTEx) dataset, along with published single-cell RNA-seq datasets of the mouse hypothalamus, to compare the relative abundance of *Igf1r* to *Insr* mRNA in different tissues and ARC neuronal populations (38, 42, 43). In humans, *IGF1R* mRNA is ubiquitously expressed in all tissues, unlike the distribution of *INSR* mRNA, which is significantly expressed in liver and skeletal muscle. The *INSR*:*IGF1R* mRNA ratio was 1.05 for the brain and 1.53 for the hypothalamus, which suggests that IGF1 signaling is relatively more predominant in the brain as compared to other tissues which have much lower *IGF1R* expression levels compared to *INSR* (Fig. 1A). Based on single-cell RNA sequencing data from HypoMap (38), *Igf1r* mRNA was found to be expressed to about the same extent (∼20%) as *Insr* in mouse ARC neurons and ARC *Pomc* + neurons, with reduced abundance (∼7.5%) in ARC NPY/AgRP + neurons. Looking at whether *Igf1r* was co-expressed with *Insr* + neurons, we found that ∼30% of *Insr* + ARC neurons, ∼13% of *Insr* + NPY/AgRP neurons, and ∼20% of *Insr* + POMC neurons expressed *Igf1r* (Fig. 1B and 1C). The same pattern of *Igf1r* and *Insr* expression is observed in human ARC NPY/AgRP and POMC neurons, although the baseline levels of these 2 receptors differ from those in mice (44). These results highlight that specific ARC NPY/AgRP and POMC neurons have the capacity to respond to IGF1 and a proportion of these are receptive to both IGF1 and insulin (44).

To further confirm this distribution pattern, we examined RNA-seq data taken from manually picked neurons originating from micro-dissected ARC tissues from *Npy*-GFP or *Pomc*-GFP-labeled mice (43). POMC-GFP neurons generally expressed more *Igf1r* mRNA when compared to NPY-GFP neurons (Fig. 1D and 1E). Using qPCR, we determined the relative expression of *Insr* and *Igf1r* in multiple hypothalamic models to assess their potential to respond to IGF1 and insulin. We found that the mHypoE-46 and the mHypoA-POMC/GFP1 immortalized cell lines, which represent models of NPY/AgRP and POMC neurons, respectively, had high *Igf1r* expression (Fig. 1E).

### *Igfbp3* and *Igfbp5* mRNA Are Expressed in Hypothalamic Neurons and Are Regulated by Fasting, Nutrients, and the Circadian Clock

Although circulating levels of IGF1 fluctuate in mice and humans depending on energy status, the hypothalamus and the cerebrospinal fluid are exposed to high baseline levels of IGF1 in the nanomolar range (45). Thus, fluctuations in circulating IGF1 levels may not be sufficient to produce meaningful changes in IGF1 signaling in the hypothalamus. Therefore, we aimed to address whether hypothalamic neurons can receive variable IGF1 input in physiological contexts. Variability in local IGF1 signaling can be tightly controlled by availability of IGF1R or the levels of IGF binding proteins, which generally sequester IGF1 from its receptor. We hypothesized that the levels of IGF1R or IGFBPs are modulated by physiological cues, including nutritional challenge, fasting, and the circadian clock, the same cues that regulate appetite and neuropeptide expression.

**Figure 2.**
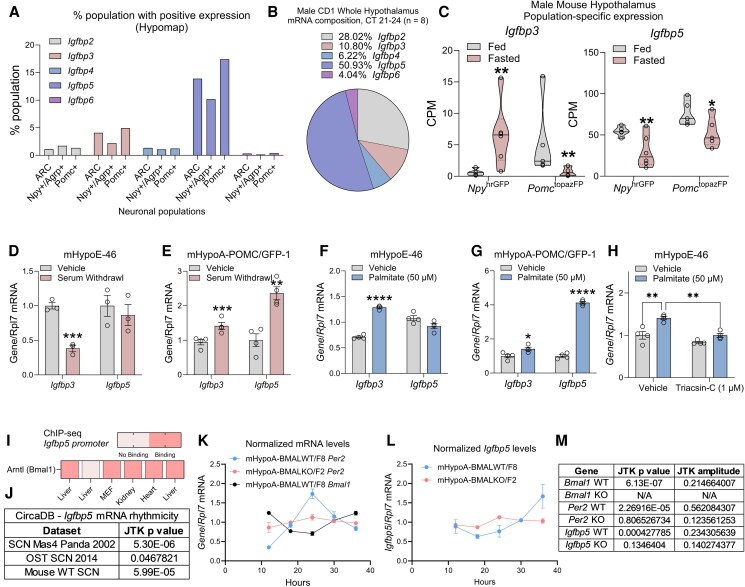
*Igfbp3* and *Igfbp5* mRNA are expressed in hypothalamic neurons and are regulated by fasting, nutrients, and the circadian clock. (A) Percentage of cells expressing *Igfbp2* through *Igfbp6* in the ARC of the hypothalamus based on HypoMap data. (B) RT-qPCR measurement of the relative contribution of *Igfbp2* through *Igfbp6* transcripts to total Igfbp2-6 expression in whole hypothalami from male CD1 mice. (C) RNA sequencing quantification of *Igfbp3* and *Igfbp5* in NPY and POMC neurons in mice after fasting. (D–E) *Igfbp3* and *Igfbp5* mRNA levels following 7 hours of FBS withdrawal in mHypoE-46 (NPY/AGRP+) neurons or mHypoA-POMC/GFP-1 (POMC+) neurons. (F–G) *Igfbp3* and *Igfbp5* mRNA levels after 24 hours of treatment with 50μM palmitate in mHypoE-46 or mHypoA-POMC/GFP-1 neurons. (H) *Igfbp3* mRNA levels after 24 hours of palmitate treatment in the presence or absence of 1μM Triacsin-C to inhibit palmitate acylation in mHypoE-46 neurons. (I) BMAL1 ChIP-seq results from various tissues, showing significant enrichment at the mouse *Igfbp5* promoter. (J) Time-course analysis of *Bmal1* and *Per2* mRNA expression in mHypoA-BMAL1/WT8 (WT) and mHypoA-BMAL1/KO2 (KO) neurons. (K) *Igfbp5* mRNA exhibits significant circadian oscillation in all 3 datasets examined in CircaDB. (L) I*gfbp5* mRNA expression in the presence and absence of BMAL1. (M) JTK cycle analysis of rhythmicity for data presented in panels K and L, performed using Nitecap. Data are presented as mean ± SEM. Statistical significance was assessed using unpaired *t* tests (D–G), and two-way ANOVA (H), with correction for multiple comparisons performed using the Tukey's method. Sample sizes (N) range from 3 to 6, as indicated in each panel. *P < .05, **P < .01, ***P < .001, ****P < .0001.

In the mouse hypothalamus, *Igfbp3* and *Igfbp5* are the only *Igfbp* in significant quantities in NPY and POMC neurons, as assessed through bulk RNA sequencing. According to HypoMap (38) single-cell RNA sequencing data, *Igfbp3* and *Igfbp5* are the most abundantly detected IGFBPs in NPY/AgRP and POMC neurons, with a higher proportion of POMC neurons expressing both, similar to the *Igf1r* expression data as previously presented (Fig. 2A). Furthermore, we measured the mRNA levels of *Igfbp2-6* in the whole hypothalamus, where abundant copies of all *Igfbps* could be detected (Ct values 21-24), with *Igfbp5* making up about 50% of all transcripts and *Igfbp3* making up about 11% of all transcripts (Fig. 2B). Given their abundance, we chose to focus on the regulation of *Igfbp3* and *Igfbp5*.

To examine whether *Igfbp3* and *Igfbp5* mRNA levels in NPY and POMC neurons are altered by fasting, a reanalysis was performed on previously published RNA sequencing data (GSE68177), which performed bulk RNA sequencing analysis on manually picked neurons originating from micro-dissected sorted arcuate *Npy*^hrGFP^ or *Pomc*^topazFP^ neurons from male transgenic mice that were fasted for 24 hours (40). Fasting did not affect *Igf1r* mRNA, but decreased *Igfbp5* in both NPY and POMC neurons, while fasting increased *Igfbp3* in NPY and decreased *Igfbp3* in POMC neurons (Fig. 2C). Next, to mimic fasting in vitro, mHypoE-46 neurons and mHypoA-POMC-GFP/1 neurons underwent serum withdrawal for 6 hours. The lack of serum decreased *Igfbp3* mRNA in mHypoE-46 neurons and increased both *Igfbp3* and *Igfbp5* mRNA in POMC-GFP/1 neurons (Fig. 2D and 2E). Although the same pattern was not observed in vivo and in vitro, both show that fasting and serum-deprivation modulates the levels of *Igfbps*. The effects of nutrients on IGFBP expression were next assessed, notably focusing on palmitate, the most abundant circulating saturated fatty acid, whose cerebrospinal fluid levels positively correlate with BMI (46). Treatment with 50µM palmitate for 24 hours increased *Igfbp3* in mHypoE-46 neurons and both *Igfbp3* and *Igfbp5* in mHypoA-POMC-GFP/1 neurons. To test whether the effects of palmitate on *Igfbp3* is caused by the buildup of palmitate metabolites, mHypoE-46 neurons were pretreated with 1µM of triacsin-C, which inhibits palmitate acylation, prior to a 24-hour treatment with 50µM palmitate (Fig. 2F and 2G). Triacsin-C blocked palmitate-mediated induction of *Igfbp3* mRNA (Fig. 2H).

Finally, given recent studies that indicate IGF1 is significantly more potent than insulin at entraining the molecular clock in hypothalamic neurons (47), we questioned whether the transcription of *Igfbps* are regulated by the circadian rhythm and BMAL1-centered circadian oscillators in the hypothalamus. To test this hypothesis, publicly available BMAL1 ChIP-seq data from various tissues were analyzed (Fig. 2I). While *Igfbp3*, *Igfbp4*, *Igfbp5*, and *Igfbp6* all display tissue-dependent binding of BMAL1 to their promoter region, only *Igfbp5* mRNA displayed circadian expression in the mouse hypothalamus based on circadian rhythm datasets in CircaDB (Fig. 2J) (48). *Igfbp5* mRNA was significantly circadian in 3 out of the 3 hypothalamus datasets, as determined by JTK cycle (Fig. 2J). To confirm the circadian nature of *Igfbp5*, this study used previously established models of NPY-expressing neurons with (mHypoA-BMALWT/F8) and without (mHypoA-BMALKO/F2) BMAL1 (25). A time-course experiment was conducted to confirm that *Bmal1* and *Per2* mRNA exhibited circadian expression in mHypoA-BMALWT/F8 neurons, and that *Per2* rhythmicity was lost in the absence of BMAL1 in the mHypoA-BMALKO/F2 neurons (Fig. 2K, 2M). In the same experiment, *Igfbp5* mRNA exhibited a circadian expression pattern that was anti-phase with *Bmal1* and in phase with *Per2*, consistent with its hypothesized regulation by the BMAL1:CLOCK complex. Notably, circadian expression of *Igfbp5* was lost and baseline expression (amplitude) of *Igfbp5* was significantly lower in the absence of BMAL1 (Fig. 2L, 2M). Together, these results demonstrate that *Igfbp5* is expressed in hypothalamic neurons and is regulated by both nutrient availability and an intact circadian clock.

### IGF1 Modulates Neuropeptide mRNA in Hypothalamic Neurons

**Figure 3.**
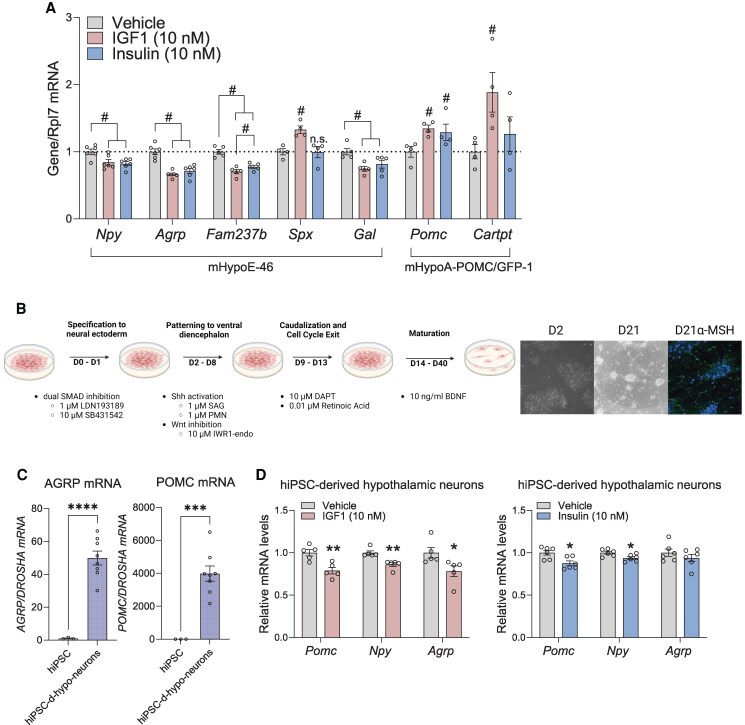
IGF1 modulated neuropeptide mRNA in hypothalamic neurons. (A) Relative mRNA levels of *Npy*, *Agrp*, *Fam237b*, *Spx*, *Gal*, *Pomc*, and *Cartpt* measured by RT-qPCR following a 6-hour treatment with 10nM IGF1 or 10nM insulin. (B) Schematic illustrating the differentiation of human induced pluripotent stem cells (iPSCs) into hypothalamic neurons, bright-field microscopy images of neuronal cultures at day 0 (D0) and day 21 (D21), and immunocytochemistry visualization of α-MSH in D21 neurons. (C) Relative *AGRP* and *POMC* mRNA levels in D21 neurons compared to D0 iPSCs. (D) Relative *AGRP* and *POMC* mRNA levels in human iPSC-derived hypothalamic neurons after a 6-hour treatment with 10nM insulin or IGF1. Data are expressed as mean ± SEM. Statistical significance was determined using one-way ANOVA with Fisher's LSD test for (A), and unpaired *t* tests for (D). Sample sizes (N) range from 3 to 8, as indicated in each panel. ^#^*P* < .05, *P* < .0001.

Since ARC neurons express *Igf1r*, and the *Igfbp* levels are regulated by physiologically relevant factors, hypothalamic neurons can thus receive dynamic IGF1 signaling. So, the next aim of our study was to examine whether IGF1 regulated neuropeptide levels in hypothalamic NPY/AgRP and POMC neurons. mHypoE-46 and mHypoA-POMC/GFP-1 neurons were treated with 10nM insulin or IGF1 for 6 hours. In mHypoE-46 neurons, both IGF1 and insulin downregulated the mRNA of *Npy*, *Agrp*, *Fam237b*, and galanin (*Gal*), which are all orexigenic neuropeptides. IGF1 resulted in a stronger downregulation of *Fam237b*. Only IGF1, but not insulin, upregulated the mRNA of the anorexigenic neuropeptide *Spexin* (*Spx*) (Fig. 3A). In mHypoA-POMC/GFP-1 neurons, both IGF1 and insulin upregulated *Pomc* mRNA, and only IGF1 upregulated the anorexigenic neuropeptide cocaine- and amphetamine-regulated transcript (*Cartpt)* mRNA (Fig. 3A). These results suggest that IGF1 acts as a potent anorexigen.

To further confirm these findings, human-origin induced pluripotent stem cells (BJ-iPSC) underwent differentiation for 21 days as per previously established protocols to generate ARC hypothalamic-like neurons (49, 50) (Fig. 3B). By day 21, iPSC-derived hypothalamic neurons were screened for the mRNA levels of *AGRP* and *POMC*, with *POMC* mRNA showing an average 4000-fold increase, demonstrating successful differentiation into *POMC*-expressing neurons (Fig. 3C). Mature neurons expressed α-MSH protein as visualized by immunocytochemistry (Fig. 3B), further confirming their identity. When human-iPSC-derived hypothalamic neurons were treated with 10 nM of recombinant IGF1 or insulin for 6 hours, IGF1 significantly downregulated *POMC*, *NPY*, and *AGRP* mRNA, while insulin modestly downregulated *POMC* and *NPY* mRNA (Fig. 3D).

### Hyperinsulinemia Promotes Cellular IGF1 Resistance in Mice and Human Hypothalamic Neurons

**Figure 4.**
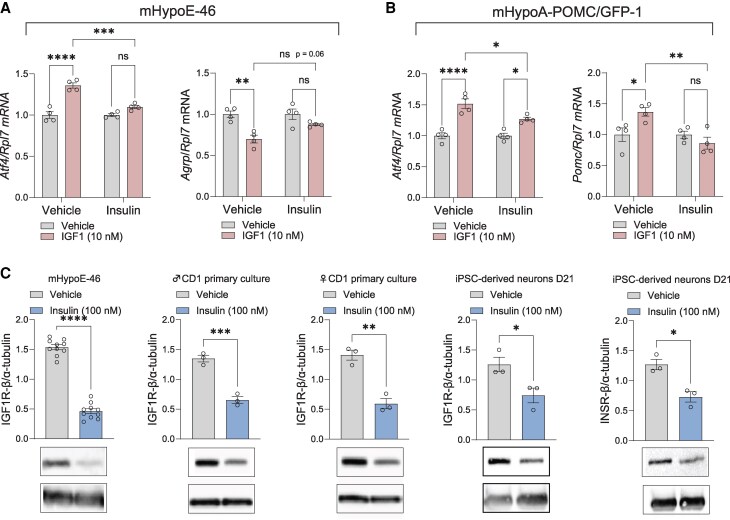
Hyperinsulinemia promoted IGF1 resistance in multiple models of hypothalamic neurons. (A) *Atf4* and *Agrp* mRNA levels in mHypoE-46 neurons following a 24-hour treatment with 100nM insulin, then an additional 6-hour treatment with 10nM IGF1. (B) *Atf4* and *Pomc* mRNA levels in mHypoA-POMC/GFP-1 neurons under the same treatment conditions as in (A). (C) IGF1R-β protein levels in mHypoE-46 neurons, as well as in primary hypothalamic cultures from male and female mice, as well as human iPSC-derived neurons (IGF1R-β and INSR), after a 24-hour treatment with 100nM insulin. Statistics were determined using two-way ANOVA for (A–B) and an unpaired, two-tailed Student *t* test for (C), with multiple-comparison adjustments made using Tukey's method. *P < .05, **P < .01, ***P < .001, ****P < .0001.

Hyperinsulinemia, as seen in prediabetic states, induces insulin resistance in neuronal models (3). Given the anorexigenic role of IGF1 in hypothalamic neurons, we questioned whether high insulin exposure would also lead to IGF1 resistance in neurons. The mHypoE-46 and mHypoA-POMC/GFP-1 neurons were treated with 100nM insulin for 24 hours, followed by 10nM IGF1 treatment for an additional 6 hours. The expression of *Atf4*, a known downstream effector of IGF1 in the hypothalamus, as well as *Agrp* and *Pomc*, the relevant neuropeptides, was subsequently measured. Insulin pretreatment essentially abrogated the ability of IGF1 to regulate *Atf4*, *Agrp*, and *Pomc* (Fig. 4A and 4B), suggesting that hyperinsulinemia induced IGF1 resistance in hypothalamic neurons. To clarify a potential mechanism, IGF1R protein levels were first measured by Western blotting. 100nM insulin treatment for 24 hours significantly reduced IGF1R protein levels in mouse mHypoE-46 neurons and primary hypothalamic neuronal cultures taken from both male and female mice (Fig. 4C). To determine whether hyperinsulinemia produced the same effect in human hypothalamic neurons, the same experiment was performed on human iPSC-derived hypothalamic neurons. Consistent with the mouse model, treatment with 100nM insulin for 24 hours significantly downregulated INSR and IGF1R protein levels (Fig. 4C).

### Hyperinsulinemia Acts Through FOXO1-mediated Transcriptional Feedback to Promote Cellular IGF1 Resistance

**Figure 5.**
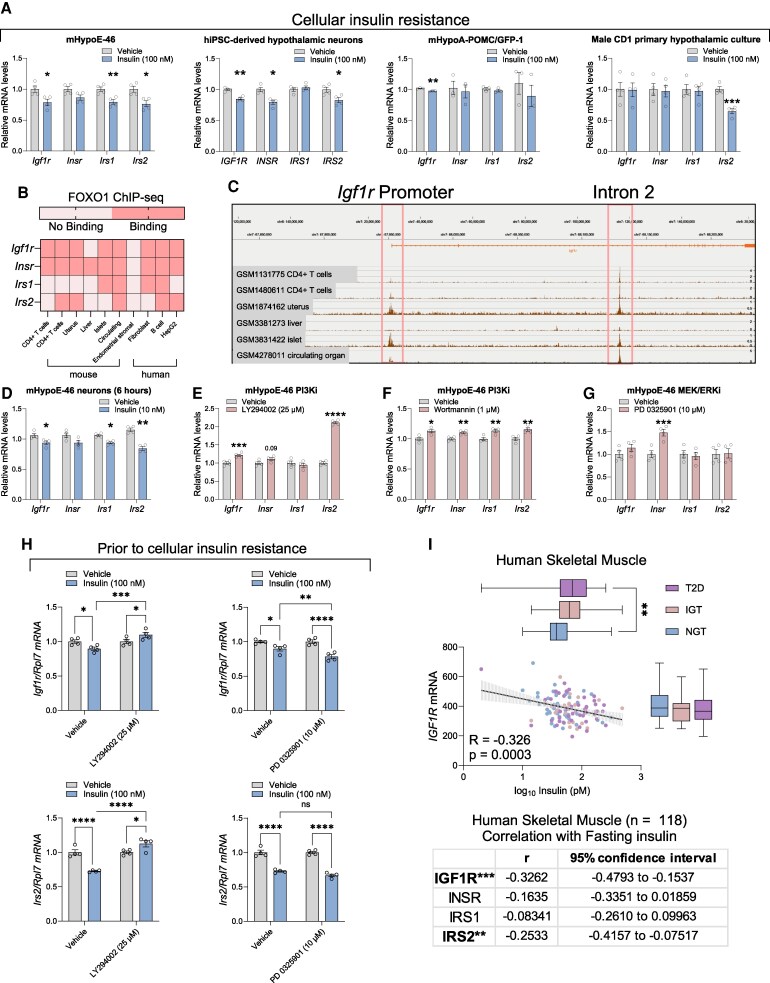
Hyperinsulinemia promoted cellular IGF1 resistance through a FOXO1-mediated transcriptional feedback system. (A) mRNA expression of *Insr*, *Igf1r*, *Irs1*, and *Irs2* in response to 24 hours treatment with 100nM insulin in mHypoE-46 neurons, human iPSC-derived POMC hypothalamic neurons, mHypoA-POMC/GFP-1 neurons, and male CD1 primary hypothalamic culture. (B) Summary of FOXO1 binding to the promoter region of mouse and human *Igf1r*, *Insr*, *Irs1*, and *Irs2*, based on public FOXO1 ChIP-seq data. (C) Visualization of FOXO1 binding to the *Igf1r* promoter and intron 2. (D) mRNA levels of *Insr*, *Igf1r*, *Irs1*, and *Irs2* in mHypoE-46 neurons after 6 hours treatment with 10nM insulin. (E-G) mRNA expression of *Insr*, *Igf1r*, *Irs1*, and *Irs2* in mHypoE-46 neurons after a 24-hour treatment with (E) 25µM LY294002 or (F) 1µM Wortmannin and (G) 8 hours of 10 µM PD 0325001. (H) *Igf1r* and *Irs2* mRNA after 6 hours of 100nM insulin treatment, with or without pretreatment with 10µM PD 0325001 or 25µM of LY294002. (I) mRNA expression of *Igf1r* in human skeletal muscle correlated with fasting circulating insulin level as well as Pearson correlation of *IGF1R*, *INSR*, *IRS1*, and *IRS2* with fasting insulin levels in human skeletal muscle. Data is expressed as mean ± SEM. Statistics in (A), (D-G) were determined using an unpaired, two-tailed Student *t* test, and in (I) using two-way ANOVA with a Fisher LSD test: *P < .05, **P < .01, ***P < .001, ****P < .0001.

To determine the mechanism by which high insulin downregulates IGF1R, we investigated the transcriptional regulation of *Igf1r* and its downstream effectors by insulin. The 100nM insulin treatment for 24 hours downregulated *Igf1r*, *Irs1*, and *Irs2* mRNA in mHypoE-46 neurons, and similarly, *IGF1R*, *INSR*, and *IRS2* mRNA in human iPSC-derived neurons (Fig. 5A). *Igf1r* was also downregulated in mHypoA-POMC/GFP neurons and *Irs2* was significantly decreased in male primary hypothalamic cultures (Fig. 5A). In mHypoE-46 neurons, treatment with 10nM insulin for 6 hours, which is prior to the development of cellular insulin resistance (51), still resulted in significant downregulation of *Igf1r*, *Irs1*, and *Irs2* (Fig. 5D). These observations suggest that decreases in mRNA levels partially account for the hyperinsulinemia-mediated repression of IGF1R protein, although additional mechanisms may be involved in primary culture, which represents a heterogeneous population of neurons. Notably, a consistent reduction in *Irs2* mRNA was observed across the cell models.

As insulin signaling is mediated partially through the PI3K/FOXO1 signaling cascade (3), bioinformatic analysis was performed to examine Foxo1/FOXO1 binding to the promoters of *Igf1r*, *Insr*, *Irs1*, and *Irs2* in 6 mouse and 4 human Foxo1/FOXO1 ChIP-seq datasets (32, 34-37, 52). Significant Foxo1/FOXO1 binding was detected at the *Igf1r* promoter in 8 of the 10 datasets, at the *Insr* promoter in 9 of the 10 datasets, at the *Irs1* promoter in 4 of the 10 datasets, and at the *Irs2* promoter in 5 of the 10 datasets. These findings suggested direct FOXO1-mediated regulation of these genes (Fig. 5B-5C).

To further confirm the involvement of the PI3K/FOXO1 cascade in the regulation of *Igf1r* and *Irs2* transcription, mHypoE-46 neurons were treated with the PI3K inhibitors LY294002 (25µM) and wortmannin (1µM) for 24 hours, and the ERK inhibitor PD 0325901 (10µM) for 8 hours. Both PI3K inhibitors upregulated *Igf1r* and *Irs2* mRNA levels, whereas only wortmannin increased *Insr* and *Irs1* (Fig. 5E and 5F). In contrast, ERK inhibition only upregulated *Insr* without altering *Igf1r* and *Irs2* mRNA levels (Fig. 5G). To further delineate whether PI3K signaling is involved, mHypoE-46 neurons were pretreated with either LY294002 (25µM) or PD 0325901 (10 M) for 1 hour, followed by a cotreatment with 100nM insulin for 6 hours. LY294002, but not PD 0325901, blocked insulin-mediated repression of *Igf1r* and *Irs2* (Fig. 5H), further demonstrating that hyperinsulinemia reduces *Igf1r* through PI3K signaling.

To determine whether IGF1 resistance may occur in other tissues, we accessed an analysis on muscle transcriptomic profiles from a study by Gallagher et al (39). The mRNA levels of *IGF1R*, *INSR*, *IRS1*, and *IRS2* were correlated with fasting levels of circulating insulin in the same participants. *IGF1R* and *IRS2* mRNA levels were negatively correlated with fasting insulin in skeletal muscle (Figs. 5I). These results collectively suggested that the transcription of *Igf1r* and *Irs2* is regulated by the PI3K/FOXO1 signaling cascade, rather than the ERK cascade, and hyperinsulinemia promotes IGF1 resistance, at least partially, through the downregulation of *Igf1r* and *Irs2* mRNA levels.

## Discussion

In this study, we demonstrated that IGF1 alters neuropeptide expression in hypothalamic neurons similar to that of insulin signaling, with an overall anorexigenic profile. We found that IGFBP5 is modulated by both fasting and circadian rhythms, suggesting a mechanism by which IGF1 signaling is regulated independently of receptor expression. Furthermore, our results indicated that anorexigenic IGF1 signaling occurs within the hypothalamus and that hyperinsulinemia-induced insulin resistance coincided with IGF1 resistance, potentially revealing a new mechanism contributing to obesity.

### IGF1 as a Signal of Energy Status

Similar to insulin, central IGF1 improved insulin sensitivity (53, 54). However, it has been difficult to assess the effects of IGF1 on energy homeostasis, given that whole-body or tissue-specific knockouts lead to growth retardation, which makes it difficult to perform glucose and insulin tolerance tests (13). On the other hand, its role in appetite regulation is less appreciated in the current literature on brain insulin and IGF1 signaling (13, 55). IGF1 fits the criteria of a hormone that signals to the hypothalamus about the energy status of the body: (i) it reaches the hypothalamus as a circulating factor, and (ii) its circulating levels change depending on energy status (eg, during fasting). This study provides evidence that hypothalamic IGF1 signaling can be modulated by nutrient availability and by circadian rhythms, and that IGF1 can regulate neuropeptide levels in hypothalamic neurons.

In addition to the recent results showing that IGF1 activates neuronal AMPK signaling, as well as protecting mitochondrial function, we demonstrated that IGF1 regulated neuropeptide expression in hypothalamic neurons and can be considered anorexigenic (56, 57). However, in contrast to previous findings in mouse neurons from our lab and others, this study indicated that acute insulin and IGF1 treatments reduced *POMC* mRNA levels in human iPSC-derived hypothalamic neurons. This unexpected result warrants further investigation to determine whether it stems from the current differentiation protocol or reflects an inherent difference between mouse and human POMC neurons; nonetheless, IGF1 and insulin also downregulated *NPY* and *AGRP* mRNA, as expected, which aligns with the mouse models. While we report that insulin and IGF1 have varying potencies in the regulation of neuropeptides, additional studies are required to study the differences in downstream signaling that may have caused the observed differences.

The insulin receptor and the insulin-like growth factor 1 receptor (IGF1R) share high sequence homology, so both insulin and IGF-1 can bind to and activate each other's receptors, albeit with significantly reduced affinity (up to 50-fold) (58). Further complicating the distinction between insulin- and IGF-1-mediated effects, there are hybrid receptors, composed of one insulin receptor α/β heterodimer and one IGF1R α/β heterodimer (59). Despite this overlap, IGF1 and insulin vary in how potently they affect gene expression in hypothalamic neurons and other models (12, 47, 56). This study identified that a substantial number of ARC neurons express high levels of IGF1R, making them likely targets of IGF1. Among these, POMC neurons may play a more significant role in hypothalamic IGF1 response compared to NPY neurons due to their more pronounced IGF1R expression. The estimated number of *Igf1r*+ neurons were confirmed using data obtained reported by Lam et al (2017), where an estimated 17% of ARC POMC neurons are *Igf1r*+ (data not shown) (60). Additionally, a distinct subpopulation of ARC neurons exhibited significantly higher *Igf1r* than *Insr* levels, suggesting that these cells may be particularly responsive to IGF1. These conclusions hold true when examining recently published single-cell RNA sequencing data of the human hypothalamus. Beyond the ARC, other hypothalamic regions may also respond to IGF1. For instance, corticotropin-releasing hormone (CRH) and oxytocin neurons in the paraventricular nucleus (PVN) express high levels of *Igf1r* mRNA, suggesting that these areas may also contribute to the effects of IGF1 on food intake (43).

### Regulation of Hypothalamic IGF1 Signaling

As shown, *Igfbp3* and *Igfbp5* are highly expressed in the hypothalamus as well as in a subset of NPY/AgRP and POMC neurons. IGFBPs are an integral part of IGF1 physiology. Only a fraction of total IGF1 is free IGF1, and this availability is tightly controlled by IGFBPs (61). An alternative perspective is that IGFBPs may keep a high concentration of IGF1 near the receptor while simultaneously constraining it. This would prevent maximal stimulation (and potential desensitization), maintaining a steady, submaximal level of receptor activation. The intricacies of IGFBP-IGF1 signaling interactions have not been examined in hypothalamic neurons and warrant further investigation in the future. The levels of IGFBPs may regulate appetite directly by regulating the availability of IGF1, which can be investigated in future studies by the central administration of IGFBP3 and IGFBP5 in vivo. In mice, intracerebroventricular administration of IGFBP3 significantly decreased peripheral glucose uptake during an insulin clamp, whereas intracerebroventricular administration of IGF1 resulted in increased peripheral glucose uptake (53, 54).

The levels of *Igfbp3* and *Igfbp5* may be pathologically regulated by palmitate and high-fat diet (HFD) feeding. Palmitate levels in the CSF positively correlate with BMI in humans (46), and our findings indicate that palmitate disrupts hypothalamic neurons through the accumulation of toxic metabolites. In this study, inhibiting long-chain fatty acyl-CoA synthetase blocked the palmitate-mediated upregulation of *Igfbp3*. Additionally, HFD feeding increased *Igfbp3* and *Igfbp5* mRNA levels in POMC-GFP neurons of the hypothalamus (*P* < .05, data not shown) (62). HFD feeding also increases *Igfbp5* mRNA in the whole hypothalamus (63).

Circadian rhythms play an important role in the regulation of diurnal feeding patterns (64). Our study showed that the BMAL1-centered circadian oscillation system regulated expression of *Igfbp5*, which peaks during nonfeeding times in animals. However, it remains unclear whether this rhythm contributes directly to appetite regulation or functions as a counterbalancing mechanism. Given that IGF1 specifically entrains circadian rhythms in hypothalamic neurons and is about 200 times more potent than that of insulin, the rhythmic expression of *Igfbp5* may also function to solely fine-tune the circadian rhythm by allowing for rhythmic IGF1 sequestering and release (47). This may also explain why nutrient intake and fasting significantly alters *Igfbp* expression in hypothalamic neurons, potentially serving as a mechanism to synchronize the clock. Given our findings, the discrepancies observed in animals administered IGF1 intracerebroventricularly may be partly attributable to the cyclical regulation of IGF1 signaling throughout the day, influenced by both nutrient status and the clock-mediated regulation of its binding proteins (15, 16).

### Hyperinsulinemia Promotes Cellular IGF1 Resistance

A feature of type 2 diabetes is the development of insulin resistance across multiple tissues, due to mechanisms such the buildup of ectopic lipids or increased inflammation. To compensate for the resistance, pancreatic beta cells secrete more insulin, leading to hyperinsulinemia, or high levels of circulating insulin (65, 66). While hyperinsulinemia is often viewed as a compensatory mechanism, it promotes metabolic disorders (7). Hyperinsulinemia can also drive additional cellular insulin resistance by decreasing insulin receptor (*Insr*) levels as well as through multiple post-receptor mechanisms (3, 51, 67, 68). Some mechanisms, such as the decrease in *Insr*, are shared across multiple tissues and cell models, which contributes to the multi-tissue insulin resistance observed in type 2 diabetes.

We found that hyperinsulinemia promoted hypothalamic neuronal IGF1 resistance, evidenced by reduced INSR and IGF1R protein levels in immortalized hypothalamic neurons, primary hypothalamic cultures, and human iPSC-derived hypothalamic neurons. Downregulation of IGF1R protein in our models was coupled with decreased *Igf1r* and *Irs2* mRNA and was observed in both mouse and human cells under prolonged high insulin conditions. FOXO1 ChIP-seq data supported a role for FOXO1 in regulating *Igf1r* transcription across multiple tissues. Inhibition of PI3K increased *Igf1r* levels and blocked the insulin-mediated repression of *Igf1r*, while ERK inhibition led to a more pronounced insulin-mediated *Igf1r* downregulation. These findings suggest that PI3K and ERK activation upon insulin exposure may counterbalance *Igf1r* mRNA levels at the transcriptional level. Nevertheless, additional mechanisms beyond PI3K-FOXO1 transcriptional feedback may be at play. For example, while hyperinsulinemia consistently decreased IGF1R protein levels in mouse primary cultures, *Igf1r* mRNA remained unchanged. This discrepancy could be due to population-specific effects, as not all neurons express significant amounts of *Igf1r*, and cell-specific effects may be masked in heterogeneous primary cultures. Other possible mechanisms, such as lysosomal degradation, have not been supported by our preliminary data.

Ample evidence indicates that tissue IGF1 resistance is present in both human obesity and animal models of obesity. In our study, we showed that hyperinsulinemia is a novel mechanism that contributed to the development of IGF1 resistance. In obese Zucker rats, elevated IGF1 coincides with insulin resistance, and in type 2 diabetic humans, the effects of IGF1 on free fatty acid (FFA) and glucose metabolism are diminished (69-73). Moreover, HFD-fed or obese mice and rats exhibit reduced muscle IGF1 sensitivity (70, 74). Insulin resistance in the human brain affected by Alzheimer disease is also associated with brain IGF1 resistance (75). Based on the ChIP-seq data, the same FOXO1 feedback loop influencing *Igf1r*, *Insr*, *Irs1*, and *Irs2* may be present in other tissues experiencing hyperinsulinemia conditions. We also uncovered that mRNA levels of IGF1R and IRS2 in muscle are inversely associated with fasting insulin levels in humans. Hyperinsulinemia may thus represent a novel mechanism underlying decreased muscle IGF1 sensitivity observed in obese and diabetic rats and humans (70, 72-74). Given the strong metabolic effects of IGF1 and the established links between its signaling components in conditions like obesity and diabetes, disrupted IGF1 signaling may also contribute to the development of obesity-related comorbidities (76).

## Conclusion

**Figure 6.**
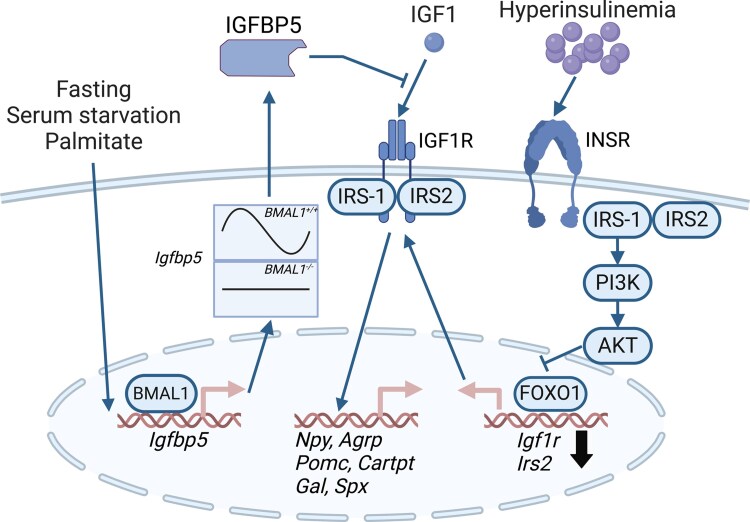
Overview of findings—hypothalamic IGF1 signaling can be dynamically regulated. *Igfbp3* and *Igfbp5* are expressed in hypothalamic neurons and are modulated by physiological cues such as fasting, nutrient availability, and circadian rhythms. IGF1 regulated neuropeptide expression in these neurons, with an overall anorexigenic profile. Hyperinsulinemia induced IGF1 resistance in hypothalamic neurons by downregulating IGF1R and insulin receptor substrate 2 (IRS2) through the phosphoinositide 3-kinase (PI3K)-FOXO1 signaling pathway.

We demonstrated that hypothalamic neurons in the ARC, specifically NPY/AgRP and POMC neurons, express significant levels of the IGF1R and responded to IGF1 signaling (Fig. 6). This study also found that hypothalamic IGF1 signaling can be dynamically regulated. *Igfbp3* and *Igfbp5* are expressed in hypothalamic neurons and are modulated by physiological cues such as fasting, nutrient availability, and circadian rhythms. IGF1 regulates neuropeptide expression in these neurons, with an overall anorexigenic profile. Importantly, this study uncovered that hyperinsulinemia induces IGF1 resistance in hypothalamic neurons by downregulating IGF1R and IRS2 through the PI3K-FOXO1 signaling pathway. This suggests that hyperinsulinemia may be a mechanism that leads to the observed tissue IGF1 resistance in human obesity and type 2 diabetes, in addition to the more characterized insulin resistance. Our study highlights the intricate interplay between insulin and IGF1 signaling in the hypothalamus and suggests that targeting IGF1 resistance may offer new therapeutic avenues for treating metabolic disorders associated with hyperinsulinemia.

## Data Availability

Data will be made available on request.

## Abbreviations

AgRP: Agouti-related peptide
ARC: arcuate nucleus
DMEM: Dulbecco’s Modified Eagle Medium
FBS: fetal bovine serum
FOXO1: forkhead box O1
HFD: high-fat diet
IGF1: insulin-like growth factor 1
IGF1R: insulin-like growth factor 1 receptor
IGFBP: insulin-like growth factor binding protein
INSR: insulin receptor
iPSC: induced pluripotent stem cell
KO: knockout
α-MSH: alpha melanocyte stimulating hormone
NEAA: non-essential amino acids
NPY: neuropeptide Y
PBS: phosphate-buffered saline
PI3K: phosphoinositide 3-kinase
POMC: pro-opiomelanocortin
RT-qPCR: reverse transcription–quantitative polymerase chain reaction
TBS-T: Tris-buffered saline with Tween 20
WT: wild-type
